# Does Institutional Social Insurance Cause the Abandonment of Cultivated Land? Evidence from Rural China

**DOI:** 10.3390/ijerph19031117

**Published:** 2022-01-20

**Authors:** Rui Min, Hongxin Yang, Xu Mo, Yanbin Qi, Dingde Xu, Xin Deng

**Affiliations:** 1 College of Economics, Sichuan Agricultural University, Chengdu 611130, China; 201906609@stu.sicau.edu.cn (R.M.); yanghongxin@stu.sicau.edu.cn (H.Y.); 201908039@stu.sicau.edu.cn (X.M.); qybin@sicau.edu.cn (Y.Q.); 2 College of Management, Sichuan Agricultural University, Chengdu 611130, China; dingdexu@sicau.edu.cn

**Keywords:** cultivated land abandonment, institutional social insurance, land social insurance function, farmer, rural China

## Abstract

At present, the world’s countryside needs to be revitalized urgently, and cultivated land is the critical factor in promoting the countryside’s revitalization. The reduction of uncultivated land contributes to the efficient use of rural land resources, contributing to global rural revitalization. This study uses data from 3938 Chinese peasant households conducted in 2014 and the OLS method to investigate the quantitative impact of institutional social insurance on cultivated land abandonment. The empirical results point to the following conclusions: (1) Institutional social insurance will weaken the social insurance function of land, which will lead to the generation of peasant households’ cultivated land abandonment. More specifically, for every 1% increase in the level of institutional social insurance of peasant households, the area of cultivated land abandoned increased by 0.002 mu; (2) The influence of institutional social insurance on peasant households’ cultivated land abandonment is heterogeneous, that is, endowment insurance can weaken the social insurance function of land more than medical insurance, and households with a higher proportion of pensioners are more likely to abandon cultivated land. This study’s conclusions may help understand the relationship between institutional and non-institutional social insurance and provide a reference for the effective use of cultivated land resources in the global rural revitalization.

## 1. Introduction

In the long course of history, land carries the dual functions of agricultural production and social insurance [1,2]. Land is not only one of the most essential factors in agricultural production, but one of the means of smoothing the risk for peasant households. In particular, land plays a vital role in rural social insurance systems in developing countries that have long lacked institutional insurance. On the one hand, land is a means of production that can bring about stable income to alleviate economic risks. Borooah et al. [3] used data from India to conclude that farmers who own land earn nearly 60% more than those who do not. On the other hand, older migrant workers who have returned to the countryside will still depend on cultivated land for their livelihoods [4,5]. Deng et al. [6] and Xu et al. [7] also found that land is an important livelihood asset for peasant households, most of whom depend on it for their livelihoods. Therefore, land ownership is one of the most important sustainable social insurance strategies for peasant households in developing countries.

However, in recent years, the phenomenon of cultivated land abandonment has become more prominent. For example, in some less developed regions of Europe and Japan, large areas of cultivated land have been abandoned. In Latvia, its national agricultural land area decreased from 3.679 million ha (57% of the total land area) in 1935 to 2.4 million ha (37% of the total land area) in 2013 [8]. As the world’s largest developing country, China’s cultivated land abandonment phenomenon cannot be ignored in this global trend. Based on national sample survey data, Xu et al. [9] pointed out that about 12% of rural households in rural China abandoned their cultivated land in 2013. Especially in the less developed mountainous rural areas, about 15% of peasant households have abandoned their cultivated land [10]. The abandonment of cultivated land may lead to soil erosion [11,12], which will reduce the quality of cultivated land and weaken the social insurance function of cultivated land. Thus, it is necessary to investigate the driving factors of cultivated land abandonment from the social insurance perspective.

In fact, with the development of society, the rural institutional social insurance system is improving gradually. For example, China launched a pilot project on the New Rural Pension Scheme in September 2009, rolling it out in rural areas in 2012. By 2016, 150 million people had started receiving pensions. In addition, the Chinese government has simultaneously promoted the New Rural Cooperative Medical Scheme, which covered 99 percent of rural residents by 2016. Compared with land’s non-institutional social insurance function, the New Rural Pension Scheme and the New Rural Cooperative Medical Scheme carry the institutional social insurance function. At the same time, rural residents also began to purchase commercial insurance to deal with potential risks. Accordingly, quantitative evidence is urgently needed to test whether the institutional social insurance will weaken the non-institutional social insurance, resulting in institutional social insurance leading to rural cultivated land abandonment.

Moreover, the world’s countryside needs to be revitalized [13], and cultivated land is an essential factor for rural revitalization promotion [14,15,16,17]. As the largest developing country globally, China is actively implementing the practice of revitalizing the countryside, with an urgent need to understand China’s land-use transition with a new perspective [18,19]. Cultivated land abandonment is a form of current rural land-use transition. At the same time, China’s rural institutional social insurance system is gradually improving. Therefore, taking China’s rural areas as an example to discuss the quantitative impact of institutional social insurance on peasant households’ cultivated land abandonment is helpful to know the unintended impact of institutional social insurance and more conducive to the effective use of rural cultivated land resources to further promote global rural revitalization. Therefore, the scientific questions of this study are:

(1) Whether the institutional social insurance affect abandonment of cultivated land? And what is the magnitude of quantitative impact?

(2) Which kind of institutional social insurance is more likely to cause peasant households to abandon cultivated land?

## 2. Theoretical Analysis

As a type of non-institutional social insurance, land ownership is one way to smooth income risk. However, with the introduction of social insurance policies such as the New Rural Pension Scheme and New Rural Cooperative Medical Scheme, the role of land as social insurance and a means of production is being weakened. As a result, farmers are less dependent on land [20].

Institutional social insurance weakens the social insurance function of the land. Compared with those insured households, land has a more robust social insurance function for non-insured households [21]. Considering the New Rural Pension Scheme, for example, Yahui et al. [22] found that the participation rate of the New Rural Pension Scheme in rural areas increased from 25.87% in 2011 to 80.85% in 2015. As the participation rate of land circulation increased from 11.56% to 24.04%, the rural land circulation rate increased by approximately 13%, and Fang and Guang [23] found that farmers with both New Rural Pension and commercial endowment insurance have significantly higher land circulation willingness than those with only one or no insurance means. The above indicates that the New Rural Pension Scheme weakens the endowment insurance function of cultivated land and makes peasant households’ dependence on cultivated land eased. In addition, with improved farmers’ insurance participation, the social insurance function of land for farmers will further decline. Xu [24] also studied the effects of institutional social insurance on peasant households with and without the elderly. The study reported that for families with or without elderly people, the New Rural Pension Scheme improves the expected level or increased the living welfare of the elderly, replacing the endowment insurance function of the land.

Similarly, the New Rural Cooperative Medical Scheme also has a partial substitution effect on the social insurance function of land, and Zhang et al. [25] found that the New Rural Cooperative Medical Scheme can significantly improve the health status of insured farmers and improve their willingness to land circulation. In addition, by analyzing survey data of peasant households in different regions of Beijing, Zhang et al. [26] concluded that the level of economic development would affect the dependence of peasant households on the social insurance function of the land. Reportedly, the higher the level of an area’s development, the greater the degree of the area’s substitution of land social insurance functions by other social insurance.

Additionally, institutional social insurance has accelerated the separation of farmers from agricultural production. In agricultural production participation data, Filho [27] reported that the rural pension reform significantly reduced the probability and time of the local elderly participating in agricultural production according to the data of Cuba. Bertrand et al. [28] and Juarez [29] found that once older people got their pension, the labor supply of their adult children in agricultural production would decrease significantly, among which the labor supply of male adult children and the eldest son would decrease more obviously. Li et al. [30] used Chinese data to investigate the impact of the New Rural Cooperative Medical Scheme on the endowment insurance and the elderly labor supply, reporting that the New Rural Cooperative Medical Scheme could significantly reduce the labor participation rate and time of the elderly. Not only that, the implementation of institutional social insurance can provide living safeguards for the elderly [31], to share the burden of their children [30], and to a certain extent, promote their children to go out to work and engage in non-agricultural employment, accelerating the separation of farmers from agricultural production [32].

In the process of improving institutional social insurance, the function of institutional social insurance will gradually replace the social insurance function of land for peasant households. It will change the behavior of farmers to use the land to a certain extent, which will lead to the decline of the social insurance function of cultivated land. When the social insurance function of land drops sufficiently, it will be abandoned.

Finally, institutional social insurance weakens the social insurance function of land, making farmers less appreciative of their land and eventually leading to abandonment.

## 3. Data, Variables, and Method

### 3.1. Data

In 2017, China first proposed implementing the rural revitalization strategy to promote the coordinated development of urban and rural areas [33]. By discussing the impact of institutional social insurance on Chinese cultivated land abandonment, it is helpful to understand the relationship between institutional social insurance and non-institutional social insurance. Equally, it provides a practical basis for establishing and improving the rural social insurance system and then promoting the rural revitalization strategy. This study uses data from the China Labor Dynamics Survey 2014 (CLDS2014) released by the Social Science Survey Center of Sun Yat-sen University in 2016, accessed from the following website: http://css.sysu.edu.cn (26 January 2017). According to Hao and Liang [34], Deng et al. [6], Deng et al. [16], and Deng et al. [35], CLDS2014 is the survey conducted in 2014 to understand the situation of peasant households in 2013, the CLDS2014 covers 29 provinces in China and is a large-scale interdisciplinary longitudinal survey that covers the political, economic, and social development of communities, as well as family information such as the demographic structure, property and income, and production.

This research discusses the impact of insurance on peasant households’ cultivated land abandonment, consequently, urban households are not included in this study. Farmers within the dataset with no information on their family members were also deleted to improve study accuracy. Finally, after data collation, this study included 3938 Chinese peasant household survey data for analysis.

### 3.2. Variables

#### 3.2.1. Dependent Variables

This study investigates the quantitative impact of institutional social insurance on cultivated land abandonment. Referring to the studies of Deng et al. [16], Xu et al. [36], and Xu et al. [9], this study selects the area of cultivated land abandoned by peasant households as the dependent variable. CLDS2014 data were collected in 2013, and abandoned cultivated land is defined as cultivated land which does not receive any factor input (e.g., labor, seeds, and fertilizer and seed) from peasant households in 2013. A distinction needs to be made here between cultivated land abandonment and fallow. In China, fallow is a cultivated land protection project implemented in specific areas (e.g., saline-alkali land, groundwater overdrawn area). Unlike cultivated land abandonment, fallow does not mean the cessation of any factor input. In contrast, fallow requires peasant households to invest labor in managing the cultivated land. Thus, the calculation formula of the area of abandoned cultivated land is:

(1) $$FA_{i}=\sum_{k=1}^{n}area_{ik}$$

where, $FA_{i}$ represents the area of cultivated land abandoned by peasant household *i* in 2013; $area_{ik}$ represents the area of cultivated land *k* abandoned by peasant households *i*.

#### 3.2.2. Focus Variables

The focus variable of this study is the level of institutional social insurance of peasant households (Social insurance). Consider that m, a member of peasant household *i*, has social endowment insurance or medical insurance, then m, a member of peasant household *i*, has institutional social insurance. The following formulas can measure this variable:

(2) $$Social\;insurance_{i}=\frac{Number\;of\;social\;insurance_{i}\;}{Total\;of\;family\;members_{i}}\times 100\%$$

(3) $$Medical\;insurance_{i}=\frac{Number\;of\;medical\;insurance_{i}\;}{Total\;of\;family\;members_{i}}\times 100\%$$

(4) $$Endowment\;insurance_{i}=\frac{Number\;of\;endowment\;insurance_{i}\;}{Total\;of\;family\;members_{i}}\times 100\%$$

where medical insurance belongs to institutional social insurance, which means compensating the insured for the economic loss caused by the risk of illness; endowment insurance belongs to institutional social insurance, which means the insured can obtain basic living security after quitting the labor post. In 2013, Chinese farmers can decide whether to buy medical insurance or endowment insurance. $Social\;insurance_{i}$ represents the institutional social insurance level of peasant households *i*; $Medical\;insurance_{i}$ represents the level of institutional medical insurance of peasant households *i*; $Endowment\;insurance_{i}$ represents the level of institutional endowment insurance of peasant households *i*; $Number\;of\;social\;insurance_{i}$, $Number\;of\;medical\;insurance_{i}$, and $Number\;of\;endowment\;insurance_{i}$ represents the number of members with institutional social insurance in peasant household *i*, the number of members with institutional medical insurance in peasant household *i*, and the number of members with institutional endowment insurance in peasant household *i*; $Total\;of\;family\;members_{i}$ represents the total number of members in peasant household *i*.

#### 3.2.3. Control Variables

Referring to the studies of Avram et al. [37], Deng et al. [38], Xu et al. [9], Huang et al. [39], Deng et al. [15], Benvenuto et al. [40], and Ma and Zhu [41], this study uses household head variables (e.g., age and education level), family variables (e.g., the total area of cultivated land, family education and family assets), land variables (e.g., land ownership and land quality), and region variables (e.g., population density, distance, and geographical location) as control variables. The details of the control variables are listed in Table 1.

### 3.3. Method

This study aims to investigate the quantitative impact of institutional social insurance on cultivated land abandonment. The dependent variable is the abandoned area of peasant households’ cultivated land. Thus, this study constructs the following econometric model for research:

(5) $$FA_{ip}=\beta_{0}+\beta_{1}Social\;insurance_{ip}+\gamma Control+\pi_{p}+\varepsilon_{ip}$$

where subscripts *i* and *p* represent peasant household *i* and province *p* respectively; *FA* represents the abandoned area of cultivated land; Social insurance represents the level of institutional social insurance; Control represents a series of control variables; $\beta_{0}$, $\beta_{1}$ and γ represents the parameters to be estimated; π represents the province dummy variable, and ε represents a random error term.

## 4. Results

### 4.1. The Influence of Institutional Social Insurance on Cultivated Land Abandonment

The estimated results of institutional social insurance impact on peasant households’ cultivated land abandonment are reported in Table 2. Model (1) mainly investigates the impact of institutional social insurance on cultivated land abandonment when only considering the situation of the peasant household. The regression results of Model (1) show that the coefficient in front of institutional social insurance (Social insurance) is positive, indicating that institutional social insurance significantly promotes cultivated land abandonment. Based on Model (1), Model (2) included external factor variables such as the proportion of the urban population in the total population in the sample County (Urbanization) to investigate whether external factors bias the conclusions from Model (1). Regression analysis of Model (2) shows persistently that the impact of institutional social insurance on cultivated land abandonment is still positive. It can be concluded that increasing institutional insurance levels will lead to expanding the levels of cultivated land abandonment. Such an outcome is less affected by external factors such as the proportion of the urban population in the total population in the sample counties. Specifically, in Model (2), the coefficient in front of social insurance is 0.002, which is significant at the level of 10%, indicating that the abandoned area of cultivated land increases by 0.002 Mu every 1% increase in the level of institutional social insurance of peasant households.

According to the descriptive statistics in Table 1, the mean of the social insurance variable is 32.26, which means that there are both those who are insured and those who are not. An endogeneity between the propensity to get social insurance and cultivated land abandonment behavior may exist. Thus, this study uses the method of the instrumental variable to solve the endogeneity problem. Referring to the studies of Sampson and Perry [42], Zimmerman [43], and Wolske et al. [44], who believed that peer behavior influences individual decision making, thus, this study defines the instrumental variable as the average social insurance of other farmers in the same village. Model (3) of Table 2 reports the estimated results by the method of the instrumental variable. Model (3) indicates that institutional social insurance still has a significant positive impact on cultivated land abandonment after focusing on endogeneity.

According to the descriptive statistics in Table 1, the mean of the social insurance variable is 32.26, which means that there are both those who are insured and those who are not. An endogeneity between the propensity to get social insurance and cultivated land abandonment behavior may exist. Thus, this study uses the method of the instrumental variable to solve the endogeneity problem. Referring to the studies of Sampson and Perry [42], Zimmerman [43], and Wolske et al. [44], who believed that peer behavior influences individual decision making, thus, this study defines the instrumental variable as the average social insurance of other farmers in the same village. Table 3 reports the estimated results by the method of the instrumental variable.

### 4.2. Heterogeneous Impact of Institutional Social Insurance on Cultivated Land Abandonment

To further analyze the impact of different types of social security on farmers’ behavior of abandoning cultivated land, this study subdivides institutional social security into institutional medical insurance and institutional endowment insurance. The specific results are shown in Table 3.

Model (1) and Model (2) demonstrate the impact of institutional medical insurance and institutional endowment insurance on cultivated land abandonment. According to the regression results, institutional endowment insurance significantly affects peasant households’ cultivated land abandonment behavior. In contrast, institutional medical insurance has no significant impact on peasant households’ cultivated land abandonment behavior. The regression results infer that institutional endowment insurance can better replace the social insurance function of land than the institutional medical insurance so that peasant households with a high proportion of members with endowment insurance are more inclined to abandon cultivated land.

## 5. Discussion

Using rural China as a case area, this study analyzes the impact of institutional social insurance on peasant households’ cultivated land abandonment behavior and its theoretical mechanism and makes an empirical test using CLDS2014. The present study extends previous research, investigating the unexpected impact of institutional social insurance and whether institutional social insurance will lead to the abandonment of cultivated land. This study also re-examines the role and impact of institutional social insurance, considering whether gradually improving the social insurance and land circulation systems will effectively use rural cultivated land resources to reduce the abandonment of cultivated land.

Cultivated land abandonment is affected by many factors. In this study, the abandonment of cultivated land occurs primarily because the institutional social insurance weakens the social insurance function of land and reduces the role of cultivated land in peasant households. The conclusion reached by the data is that the social insurance function of land is showing a weakening trend.

Imperfect land circulation mechanisms and difficulties in land circulation are two other factors leading to cultivated land abandonment [45]. Previously, some studies showed that the implementation of institutional social insurance would increase the willingness of farmers to carry out land circulation [46] or reduce the willing rent of land [47] then can reduce cultivated land abandonment. However, the quantitative analysis in this study shows that institutional social insurance will contribute to the abandonment of cultivated land. Analysis of the data has shown the possible causes are: the influence of external environments, such as land circulation systems not functioning well, or the land development market is not trustworthy, making farmers unwilling to transfer their land. Alternatively, farmers are willing to transfer but do not transfer [48], which leads to the abandonment of cultivated land. Finally, the substitution effect of institutional social insurance on land insurance may be heterogeneous. Qin et al. [47] found that the social insurance function of land depends on the degree of dependence of peasant households on land. The substitution effect of social insurance on land mainly influences the peasant households who are highly dependent on land.

However, the present study still has some limitations that should be addressed in future research, for example:

(1) This study focuses on the quantitative impact of institutional social insurance on cultivated land abandonment. Future research can further empirically analyze how institutional social insurance substitutes the land insurance function and affects cultivated land abandonment.

(2) This study only investigated the heterogeneity of the two types of institutional social insurance, pension, and medical care. In the future, the impacts of more types of institutional social insurance or commercial insurance on peasant households’ cultivated land abandonment behavior should be investigated.

(3) China is the largest developing country with rapid development in the world. And its development experience may have some implications for other developing countries. However, China has a special land system, and whether the conclusion of this study can be applied to other developing countries needs to be further discussed in the future.

## 6. Conclusions and Policy Revelations

### 6.1. Conclusions

Based on 3938 survey data in China’s rural areas, this study quantitatively investigates the impact of institutional social insurance on peasant households’ cultivated land abandonment and draws the following conclusions:

(1) The improvement of institutional social insurance level will weaken the social insurance function of land to a certain extent, resulting in the abandonment of cultivated land by peasant households.

(2) The impact of institutional social insurance on peasant households’ cultivated land abandonment behavior is heterogeneous, and the institutional endowment insurance can replace the social insurance function of land better than the institutional medical insurance, so the peasant households with a high proportion of members of endowment insurance are more inclined to abandon the land.

### 6.2. Policy Recommendations

The above conclusions have important policy revelations for China’s rural land reform and social insurance system improvements:

(1) The study’s empirical results demonstrate that institutional social insurance will significantly lead to the abandonment of cultivated land. The peasant households with a high proportion of members with endowment insurance are more inclined to abandon the land. Therefore, while improving rural old-age care, we should also pay attention to the cultivation of the land market and improve the land circulation mechanism to avoid the abandonment of cultivated land caused by institutional social insurance.

(2) The impact of institutional social insurance on peasant households’ cultivated land abandonment behavior is heterogeneous. Institutional endowment insurance can replace the insurance function of land better than the institutional medical insurance, inferring that the rural medical insurance system development is inferior to the rural endowment insurance system. Therefore, the current New Rural Cooperative Medical Scheme system must be actively explored to break through the bottleneck of medical insurance system development and establish an efficacious and functional medical care system.

(3) To further solve cultivated land abandoned by peasant households, cultivated land must be integrated with rural revitalization and developing land reforms. At the same time, we need to strengthen the construction of agricultural infrastructure and promote agricultural modernization.

## Figures and Tables

**Table 1:** Definition and statistical results.

Variables	Definition	Mean	S.D.
Abandonment area	Area of family abandoned cultivated land (MU)	0.34	1.76
Social insurance	Proportion of family members with medical or endowment insurance in the total number (%)	32.26	22.76
Medical insurance	Proportion of family members with medical insurance in the total number (%)	31.36	22.82
Endowment insurance	Proportion of family members with endowment insurance in the total number (%)	16.88	21.33
Head age	Age of head of household (year)	52.10	11.00
Head education	1 if the household head has a high schooldiploma or above; 0 otherwise	0.11	0.32
Farm income	Proportion of agricultural income in total farm income (%)	38.59	41.54
Land size	Total cultivated land area (MU)	7.88	10.15
Family education	Proportion of members with high school education or above in the total number (%)	14.22	19.80
Family health	Proportion of healthy family members in the total number (%)	88.03	20.04
Older	1 if the elderly in the family are engaged in agricultural production;0 otherwise	0.11	0.32
Farm successor	1 if young people in the family are engaged in agricultural production;0 otherwise	0.09	0.29
Fixed assets	Present value of household fixed assets (10,000 yuan)	3.75	13.68
Agricultural assets	Present value of household agricultural assets (10,000 yuan)	0.09	0.68
Land registration	1 if the family owns a land registration certificate;0 otherwise	0.44	0.50
Land Quality	1 if the average level of family cultivated land quality is low;0 otherwise	0.03	0.18
Land irrigation	1 if Cultivated land has irrigation facilities; 0 otherwise	0.43	0.49
Urbanization	Proportion of urban population in the total population in the same sample County (%)	10.14	19.49
Population density	Density of village population (num/km^2^)	134.49	124.04
Distance	Minimum distance from the village to business center (km)	6.76	8.45
Plain	1 if the village is located on the plain; 0 otherwise	0.41	0.49
Hill	1 if the village is located in the hills; 0 otherwise	0.32	0.47
Mountain	1 if the village is located in the mountain; 0 otherwise	0.27	0.44

**Table 2:** The estimates of impacts of institutional social insurance on cultivated land abandonment.

	Model (1)	Model (2)	Model (3)
Social insurance	0.002 *	0.002 *	0.009 **
	(1.701)	(1.738)	(2.168)
Head age	0.011	0.014	0.009
	(1.057)	(1.301)	(0.857)
Head age2	−0.000	−0.000	−0.000
	(−1.346)	(−1.194)	(−0.744)
Head education	0.009	0.019	0.022
	(0.058)	(0.118)	(0.141)
Farm income	−0.001	−0.001	−0.001
	(−1.499)	(−1.216)	(−1.603)
Land size	0.029 **	0.034	0.041
	(2.338)	(1.237)	(1.375)
Family education	0.001	0.000	0.000
	(0.604)	(0.201)	(0.255)
Family health	−0.003 ***	−0.001	−0.001
	(−2.615)	(−0.817)	(−0.687)
older	−0.002	−0.037	−0.021
	(−0.016)	(−0.351)	(−0.202)
Farm successor	0.017	−0.058	−0.042
	(0.133)	(−0.442)	(−0.328)
Ln(Fixed assets)	−0.052 **	−0.042 *	−0.065 **
	(−2.217)	(−1.900)	(−2.504)
Ln(Agricultural assets)	−0.231 ***	−0.268 **	−0.332 ***
	(−2.670)	(−2.528)	(−2.625)
Land registration	−0.067	−0.067	−0.077
	(−1.392)	(−1.211)	(−1.347)
Land Quality	3.491 ***	2.946 ***	2.945 ***
	(7.909)	(7.640)	(7.646)
Land irrigation	0.185 ***	0.208 ***	0.210 ***
	(3.172)	(3.125)	(3.113)
Urbanization		−0.003 ***	−0.003 **
		(−2.710)	(−2.252)
Population density		−0.001 ***	−0.001 ***
		(−4.152)	(−3.906)
Distance		0.003	0.003
		(0.862)	(0.951)
Plain		0.276 **	0.283 **
		(2.237)	(2.271)
Hill		0.117	0.108
		(1.400)	(1.294)
Province dummies	No	Yes	Yes
ll	−7464.899	−7301.618	-
r2	0.160	0.227	0.220
N	3938	3938	3938

Note: *t* statistics in parentheses; * *p* < 0.1, ** *p* < 0.05, *** *p* < 0.01.

**Table 3:** The estimates of heterogeneous effects.

	(1)	(2)
	Model (1)	Model (2)
Medical insurance	0.002	
	(1.482)	
Endowment insurance		0.003 **
		(2.121)
Control variables	Yes	Yes
ll	−7302.096	−7300.588
r2	0.227	0.227
N	3938.000	3938.000

Note: *t* statistics in parentheses; ** *p* < 0.05.

## Data Availability

The datasets used and/or analyzed during the current study are available from the corresponding author on reasonable request.
